# Small for gestational age babies and depressive symptoms of mothers during pregnancy: Results from a birth cohort in India

**DOI:** 10.12688/wellcomeopenres.14618.3

**Published:** 2020-02-06

**Authors:** Giridhara R. Babu, G.V.S. Murthy, Yogesh Reddy, R. Deepa, A. Yamuna, S. Prafulla, Anjaly Krishnan, Eunice Lobo, Mohanbabu Rathnaiah, Sanjay Kinra

**Affiliations:** 1 Indian Institute of Public Health - Bangalore, Bengaluru, Karnataka , 560023, India; 2 The Wellcome Trust/DBT India Alliance, New Delhi, 110025, India; 3 Indian Institute of Public Health, Public Health Foundation of India, Madhapur, Hyderabad, 500033, India; 4 International Centre for Eye Health, Faculty of Infectious and Tropical Diseases, London School of Hygiene & Tropical Medicine, London, WC1E 7HT, UK; 5 Department of Psychiatry, Institute of Mental Health, University of Nottingham, Nottingham, NG7 2TU, UK; 6 Department of Non-communicable Disease Epidemiology, Faculty of Epidemiology and Population Health, London School of Hygiene & Tropical Medicine, London, WC1E 7HT, UK

**Keywords:** Small for Gestational age, low birth weight, Prenatal depression, Screening, Pregnancy, birth cohort, public hospital, Low and Middle Income Country

## Abstract

**Background**: Annually, more than a million low birthweight (LBW) is born in India, often afflicting disadvantaged families. Several studies have undertaken the association of poverty, nutritional status, and obstetric factors with LBW. Through our study, we aimed to examine the possibility of any relation between the Edinburgh Postnatal Depression Scale (EPDS) score measured during pregnancy with the incidence of babies born Small for Gestational Age (SGA).

**Methods**: Pregnant women attending the antenatal clinic at a public hospital between 14 to 32 weeks were recruited from April 2016 to Oct 2017. The EPDS was administered to assess depression through face-to-face interviews. Newborn anthropometry was performed post-delivery. For analysis, birth weight <10 percentile was classified as SGA.

**Results**: Prevalence of depressive symptoms (EPDS score >11) was 16.5% (n=108/654) in antenatal mothers. These women delivered a higher proportion of SGA babies (21.3 v/s 15.8) compared to women with no symptoms. The odds of women giving birth to a child with SGA were twice as high for women with EPDS scores >11 (adjusted OR = 2.03; 95% CI = 1.12 – 3.70) compared to the women with EPDS scores of ≤11, The EPDS 12 (Adjusted OR = 1.96; 95% CI = 1.04 – 3.69) and EPDS 13 (Adjusted OR = 2.42; 95% CI = 1.24 – 4.70) cut-off categories also proved to be a risk factor for SGA with significant p-value (0.0006 and 0.0003) and the individuals with more than 13 EPDS score is found to have the highest odds of SGA.

**Conclusions**: We found a strong association of antenatal depressive symptoms during pregnancy with SGA measured by EPDS. Thus, we recommend the implementation of timely and effective screening, diagnostic services, and evidence-based antenatal mental health services to combat SGA and further associated-metabolic syndromes.

## Introduction

Low birth weight (LBW; <2500 g), a marker of poor intrauterine growth, leads to the double burden of stunting in childhood and predisposes to obesity in adolescence ^1, 2^. The pathways triggered by LBW lead to perpetuating, independent cycles of ill health ^3, 4^. More than one million babies are born with LBW in India every year. LBW often afflicts disadvantaged families, accentuating the risk of child mortality and morbidity ^5^. Despite the high prevalence of LBW, its causes are poorly recognized. Infants with LBW comprises of preterm babies (<37 weeks gestation) or Small for Gestational Age (SGA) or both ^6^. SGA is defined as birth weight below the population-specific 10 ^th^ percentile for the gestational age. Children, who are born SGA, have several short and long-term adverse outcomes ^7– 9^.

Apart from the increased risk of mortality, infants with SGA might have a broad spectrum of adverse growth, morbidity, and developmental outcomes ^10^. Due to poor nutritional status, a range of problems from malabsorption to growth retardation can affect the growing children ^11^. The ‘thrifty phenotype' hypothesis describes that adaptive mechanisms due to child undernutrition are on the rise and result in type 2 diabetes mellitus (T2DM), which is epidemic in low- and middle-income countries (LMICs). Confronted with undernutrition as a fetus and child, the compensatory adaptive mechanism stores excess energy as fat ^12^. As a result, LBW in babies accentuates the risk of obesity, insulin resistance, cardiovascular diseases and T2DM ^13^.

Over the past several decades, program interventions to reduce LBW have mostly focused on addressing poverty, maternal nutritional status, and obstetric factors in India. However, the proportion of children with LBW has remained stagnant or reduced only minimally over this period in LMICs, such as India. The role of antepartum depression is often neglected as a determinant of SGA, despite evidence indicating that women with antepartum depression have an increased risk of having a preterm birth and LBW babies ^14^. Meta-analyses also suggest that the magnitude of this association varies with how depression is measured, country of residence and socioeconomic status ^14, 15^. Almost all the evidence on the impact of antepartum depression on LBW is from developed countries. As an exception, a study from Bangladesh has suggested an association of high Edinburgh Postnatal Depression Scale (EPDS) score in pregnant women may be associated with LBW ^16^. Also, the role of EPDS as screening criteria for antepartum depression is underexplored in most LMICs, and studies have used different cut-offs for different samples ^17^.

This study aims to examine if the relationship between the Edinburgh Postnatal Depression Scale (EPDS) score and SGA. Despite the high prevalence of SGA in LMICs such as India, the awareness of mental health problems is low. Antenatal depression in pregnancy is not routinely screened in LMICs, including whether it can be a risk factor for poor intrauterine growth. This is specifically relevant in metropolitan cities like Bangalore, which has relatively better socio-economic standards in communities compared to several other regions but continues to experience persistently high proportions of children born with SGA.

## Methods

### Study setting

**Figure 1.**
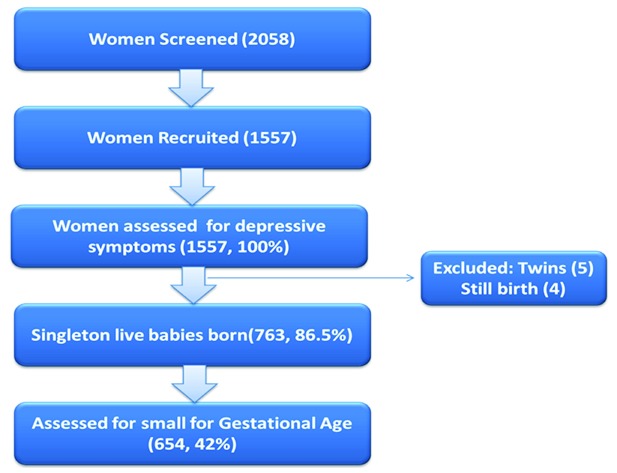
Flow diagram depicting the composition of the ongoing cohort and present study sample (n=654) from the MAASTHI birth cohort. See 18 for further details of the MAASTHI cohort.

Maternal antecedents of adiposity and studying the transgenerational role of hyperglycemia and insulin (MAASTHI) is a birth cohort established to prospectively identify risk factors in pregnancy associated with adverse infant outcomes, especially in predicting the possible risk markers of later chronic diseases ^18^. The detailed protocol of the study has been published elsewhere ^18^. Briefly, pregnant women with gestational age (GA) between 14 to 32 weeks were recruited. GA was determined by ultrasonography record and if not available, the last menstrual period was noted. In the 1557 women enrolled, 654 women who had completed follow up after delivery comprise the study sample for the present study, stillbirth and twins were excluded from the data analysis. ( Figure 1).

### Data collection

Data was collected from April 2016 to October 2017 at a secondary level public hospital. Data at baseline (second and third trimester of pregnancy) included socioeconomic conditions that included religion, education, occupation and the women's reproductive history, social support, depressive symptoms and consumption of tobacco and alcohol. EPDS tool was translated into the local language (Kannada) and then back-translated to English for accuracy. Through this, efforts were made to ensure a clear and conceptually accurate translation that was easily understood by the local population. The Questionnaire was then administered to the respondents by trained research assistants who would interview without altering the actual meaning. The response score is quantified by asking the frequency of occurrence of depressive symptoms for several days. The respondent's weight, height, Mid-upper arm circumference (MUAC), head circumference, biceps, triceps and subscapular skinfold thickness were recorded. Birth data were collected through structured interviews and anthropometric assessment by trained female research staff in the hospital. The data collection for pregnant women regarding depressive symptoms was done during the second and third trimester, and the anthropometry of the newborn was recorded between 2 to 48 hours following delivery. Several birth outcomes were assessed including the length of pregnancy, mode and place of delivery, complications during labour, live or stillbirth, birth weight, length, head, chest, waist, hip and MUAC of the newborn. Skinfold thickness was measured using Holtain callipers at biceps, triceps and subscapular sites.

### Measurements

***Assessment of antepartum depressive symptoms.*** The Edinburgh Postnatal Depression Scale (EPDS) is a widely used self-reporting questionnaire developed specifically to screen for symptoms of perinatal depression ^19, 20^. EPDS has been validated by Fernandes *et al*. for prenatal depression in South India at a cut-off of ≥13 (sensitivity = 100%, specificity = 84.90%, and AUC = 0.95) ^21^. Depressive symptoms are assessed by a 10-item scale, which determines the psychosocial stress level of pregnant women in the last seven days. Social support was measured using a questionnaire developed at St. John’s Research Institute to evaluate a broad range of social support (i.e., emotional, instrumental, informational, and appraisal) ^22^. This questionnaire has a total of 12 items, and each item is scored between 0 (definitely not enough) to 3 (definitely enough). The highest score being 36 means excellent social support and 0 meaning low social support. The scale reported an excellent value of internal consistency, as determined by Cronbach's alpha of 0.935 all variables showing a high level of consistency. Trained Research Assistants using an Android tablet administered the questionnaire; the system is programmed to generate a EPDS score in real-time, and in case the woman scored >13 she was referred to the psychiatrist at the hospital. The correlates of EPDS have internal consistency exceeding 0.8. Pregnant women were classified into two groups based on their EPDS score: 0–11, without depressive symptoms; 11+ with depressive symptoms. This 10-item scale has been translated into many different languages and validated in many countries, including India ^23^. The cutoff values of EPDS as a screening tool for antenatal depression in primary health care settings is dependent on cultural settings. For example, a cut-off EPDS score for the Spanish version of the EPDS is 8/9, and the Chinese version is 9/10 ^24^. A cutoff score of 11/12 was found to detect perinatal depression with acceptable sensitivity and specificity in Goa, India ^25^. In concurrence with this evidence, we aimed to assess the exact EPDS score cut-off value (11, 12 or 13) as a better predictor of association between antenatal depression and SGA.

***Other risk factors.*** Possible risk factors for SGA were assessed by a standardized questionnaire seeking information on women’s medical and obstetric history (parity, abortion), socio-economic and demographic characteristics (age, education, and occupation), smoking habits and alcohol consumption. The research staff measured women’s height, weight, MUAC. Skinfold thickness was measured using Holtain callipers at biceps, triceps and subscapular sites.

***Anthropometry.*** Adult anthropometry: After ensuring that the scale was placed on level ground, the research staff would view 'zero' reading. After ensuring that the respondent would remove heavy outer clothing and shoes, two readings to the nearest 10 gram were taken. Further, we used SECA 213 portable stadiometer for measuring the height to the nearest 0.1 cm. This was measured by requesting the respondent to stand straight with her feet together, ensuring the posterior surface of the head and heels was applied to the stadiometer. The head was positioned in an imaginary line joining the upper margin of the external auditory meatus and the lower border of the orbit of the eye (Frankfurt plane). The head plate of the stadiometer would then be pulled down to ensure that it rests on the crown of the head ^26^.

*Baby anthropometry*: Newborn anthropometry was performed using SECA 354 Weighing Scale and SECA 417 Infantometer. The baby was placed naked on the digital weighing scale, and readings are taken to the nearest 0.5g. For measuring infant length, the baby's head is held against the end of the head plate and the legs extended until they are flat. The footplate is brought up to the heels ensuring that feet and knees were flat, the length is recorded. Chasmors body circumference tape was used to measure the circumferences. Head circumference is measured with the baby's head on the side so that the maximum occipitofrontal circumference could be found. The tape was placed on the forehead, on the most anterior point (just above the eyebrows) and passed around the head to the most posterior part of the head making sure the maximum circumference is found. Waist circumference was taken by placing the tape around the abdomen immediately above the umbilicus, ensuring that it is horizontal and marked at the end of expiration. Chest circumference is measured by placing the tape around the chest at the level of xiphisternum, ensuring that it is placed horizontal and marked at the end of expiration. MUAC was recorded with the arm bent, allowing the measurement to be taken with the baby in its natural position. Skinfold thickness is measured on the left side of the body using the Holtain Calipers. Three readings to the nearest 0.2mm were taken unless this caused too much distress, in which case, a single measurement was taken. For triceps skinfold thickness, the tape is placed around the upper arm at the level of the mark done while measuring MUAC. With the tape in position, a horizontal line is drawn on the skin posteriorly at the level of the mark. Another vertical line is marked on this line at the most dorsal part of the upper arm. This level was determined by 'eyeballing' the mid-point. The point at which the fold is to be measured was then marked; the skin was lifted over the posterior surface of the triceps muscle, above the marked point, on a vertical line passing upward from the olecranon to the acromion. The callipers are applied below the fingers such that the marked cross was at the apex of the fold. Biceps skinfold is measured in the anterior midline of the arm over the biceps on the same level as the triceps skinfold. For subscapular skinfold thickness, the inferior angle of the scapula was identified, and the skin is marked immediately below the angle. The skinfold was picked up above the mark with the fold slightly inclined downward and laterally, in the natural cleavage of the skin. The calliper jaws are applied below the fingers, such that the marked point is at the apex of the fold ^26^.

The weight of infant was classified into percentiles based on the Indian standards for birth weights of newborns based on the sex and order of the baby ^27^. Anything less than 10 percentile was classified as SGA, between 10 to 90 ^th^ percentile was appropriate for gestational age (AGA) and greater than 90 ^th^ percentile was large for gestational age (LGA). Babies born before 37 weeks of gestation were considered premature. Other details of neonatal morbidity and hospitalization were obtained from the family members and medical records.

### Statistical analysis

We used logistic regression analysis to assess the association between SGA and EPDS score. The association with SGA was examined taking the EPDS score as a continuous as well as categorical predictors. The 3 categorical variables were formed based on the cut-off scores of 11, 12 and 13. This was adjusted for known confounders based on literature review for maternal age, religion, respondent's and husband's incomes, gravida, parity, husband's current consumption of tobacco and alcohol and respondent's sum of skinfold thickness. These variables were adjusted based on the priori information ^28– 33^. The goodness of fit of the models was assessed using the Hosmer-Lemeshow statistic. Statistical analysis was performed using Stata/IC 14.2 for Mac (Revision 19 Dec 2017, Copyright 1985–2015 StataCorp LLC) and SPSS version 23. Descriptive analysis was done for maternal and neonatal characteristics for both women with and without depressive mental symptoms.

## Results

A total of 654 pregnant mothers who completed the EPDS questionnaire were taken into consideration for analysis in the present study. The mean maternal age of the study sample at baseline was 23.6 ± 3.9 years. Mothers with depressive symptoms had lower mean social support scores compared to mothers without depressive symptoms ( Table 1). The study found that overall, 16.51% (n=108) of the antenatal mothers had depressive symptoms (EPDS score of >11).

**Table 1.** Maternal characteristics in relation to depressive symptoms during pregnancy.

Characteristic	Total [N =654]	EPDS ≤ 11 (without depressive symptoms) [N = 546]	EPDS > 11 (with depressive symptoms) [N = 108]
Age (years)	23.62 ± 3.91	23.66 ± 3.83	23.43 ± 4.31
Respondent’s income	431.50 ± 1888.46	450.92 ± 1980.19	333.33 ± 1334.31
Husband’s income	11493.85 ± 5884.02	11613.47 ± 6061.27	10893.52 ± 4878.93
Social support	24.73 ± 11.05	25.49 ± 10.65	20.88 ± 12.24
	216 (33.0)		
	255 (39.0)		
	144 (22.0)		
	33 (5.0)		
	6 (0.9)		
Religion			
* Hinduism*	280 (42.8)	245 (44.9)	35 (32.4)
* Christianity*	19 (2.9)	17 (3.1)	2 (1.9)
* Islam*	355 (54.3)	284 (52.0)	71 (65.7)
Respondent’s education			
* Illiterate*	19 (2.9)	15 (2.7)	4 (3.7)
* Primary school*	36 (5.5)	33 (6.0)	3 (2.8)
* Middle school*	113 (17.3)	88 (16.1)	25 (23.1)
* High school*	290 (44.3)	241(44.1)	49 (45.4)
* Pre-university*	153 (23.4)	136 (24.9)	17 (15.7)
* Graduate or above*	43 (6.6)	33 (6.1)	10 (9.3)
*Consanguineous Marriage*			
* Yes*	204 (31.2%)	167 (30.6)	37 (34.3)
* No*	450 (68.8%)	379 (69.4)	71 (65.7)
Kuppuswamy scale			
* Upper*	4 (0.6)	4 (0.7)	0
* Upper middle*	594 (90.8)	495 (90.7)	99 (91.7)
* Lower middle*	52 (8.0)	43 (7.9)	9 (8.3)
* Lower*	4 (0.6)	4 (0.8)	0
Gravida			
* 1*	234 (35.8)	189 (34.6)	45 (41.7)
* 2*	273 (41.7)	238 (43.6)	35 (32.4)
* 3*	114 (17.4)	93 (17.0)	21 (19.4)
* More than 3*	33 (5.1)	26 (4.7)	7 (6.5)
Parity			
* 0*	275 (42.0)	224 (41.0)	51 (47.2)
* 1*	313 (47.9)	272 (49.8)	41 (38.0)
* 2 or more*	66 (10.1)	50 (9.1)	16 (14.8)
Anaemia Status			
* Present*	300 (45.9%)	253 (46.3% )	47 (43.5% )
* Absent*	354 (54.1% )	293 (53.7% )	61 (56.5% )
Tobacco consumption among husbands			
* Yes*	295 (45.1)	230 (42.1)	65 (60.2)
* No*	359 (54.9)	316 (57.9)	43 (39.8)
Alcohol consumption among husbands			
* Yes*	91 (13.9)	68 (12.5)	23 (21.3)
* No*	563 (86.1)	478 (87.5)	85 (78.7)

*Values are presented as mean ± standard deviation or n (%); EPDS: Edinburgh Postnatal Depression Scale*

Among mothers with depressive symptoms (EPDS score >11), 43 (39.8%) mothers were below the age of 22 years. Depressive symptoms affected predominately young mothers and the symptoms decreased with increase in age of the women. The majority of the study sample comprised of Muslim women, and they were the most afflicted with depressive symptoms (65.7%), followed by mothers belonging to the Hindu religion (32.4%). Pregnant women with high school education had a high proportion of depressive symptoms (44.3%) compared to other levels of educational attainment. Among the pregnant women, the depressive symptoms in the women with first pregnancy were high (41.7%) and decreased with an increase in the number of times conceived and delivered. The results indicate that 60% of husbands of pregnant women with depressive symptoms were consuming tobacco, and 21% were drinking alcohol ( Table 1).

Women with depressive symptoms delivered a greater proportion of SGA (21.3 *vs* 15.8%) compared to women with no symptoms. While there were no major differences for normal term delivery, women with depressive symptoms had a slightly elevated proportion of caesarian section delivery (31.5 *vs* 24.2%) ( Table 2).

**Table 2.** Neonatal characteristics in relation to depressive symptoms during pregnancy.

Characteristic	Total [N =654]	EPDS ≤ 11 (without depressive symptoms) [N = 546]	EPDS >11 (with depressive symptoms) [N = 108]
Gender of baby			
* Female*	337 (51.5)	277 (50.7)	60 (55.6)
* Male*	317 (48.5)	269 (49.3)	48 (44.4)
Delivery type			
* Normal*	341 (52.1)	286 (52.4)	55 (50.9)
* Primary C-section*	166 (25.4)	132 (24.2)	34 (31.5)
* Repeated C-section*	147 (22.5)	128 (23.4)	19 (17.6)
Weight categories			
* SGA*	109 (16.7)	86 (15.8)	23 (21.3)
* AGA*	517 (79.1)	442 (81.0)	75 (69.4)
* LGA*	28 (4.3)	18 (3.3)	10 (9.3)
Premature delivery			
* Yes*	61 (9.3)	52 (9.5)	9 (8.3)
* No*	593 (90.7)	494 (90.5)	99 (91.7)

Values are presented as mean ± standard deviation or n (%); EPDS: Edinburgh Postnatal Depression Scale; C-section: caesarian delivery; SGA: small for gestational age; AGA: appropriate for gestational age; LGA: large for gestational age.

Maternal and neonatal characteristics in relation to SGA and AGA status are summarized in Table 3.

**Table 3.** Maternal and neonatal characteristics in relation to small for gestational age (SGA) babies.

Characteristic	SGA (N = 109)	AGA (N = 517)
Maternal characteristics		
*Age at the baseline*	24.12 ± 3.76	23.55 ± 3.93
* Gravida*	1.93 ± 0.80	1.93 ± 0.93
* Parity*	0.73 ± 0.56	0.68 ± 0.69
* Abortion*	0.28 ± 0.58	0.28 ± 0.56
* EPDS Score (Pregnancy)*	6.27 ± 5.71	5.73 ± 5.20
* BMI (kg/m2)*	22.67 ± 3.64	24.42 ± 4.32
Maternal anthropometric measurements		
* Weight (kg)*	52.87 ± 8.76	58.51 ± 10.79
* Height (cm)*	152.78 ± 5.77	154.77 ± 5.17
* Mid-upper arm circumference (cm)*	24.89 ± 2.96	26.15 ± 3.55
* Biceps skinfold thickness (mm)*	8.57 ± 3.38	9.59 ± 3.66
* Triceps skinfold thickness (mm)*	18.87 ± 5.30	20.59 ± 5.89
* Subscapular skinfold thickness (mm)*	15.08 ± 5.36	16.88 ± 5.78
* Sum of skinfold thickness (mm)*	42.53 ± 12.71	47.06 ± 13.73
* Gestational age at delivery (weeks)*	39.22 ± 1.14	38.65 ± 1.43
Neonatal anthropometric measurements		
* Weight (Kg)*	2.31 ± 0.23	2.80 ± 0.29
* Length (cm)*	47.29 ± 2.43	48.30 ± 2.49
* Crown-rump length (cm)*	30.69 ± 2.84	31.63 ± 3.25
* Head circumference (cm)*	32.32 ± 1.34	32.99 ± 1.37
* Chest circumference (cm)*	29.75 ± 1.82	31.17 ± 1.72
* Waist circumference (cm)*	26.45 ± 2.57	28.23 ± 2.34
* Hip circumference (cm)*	23.51 ± 5.43	25.77 ± 5.07
* Mid-upper arm circumference (cm)*	10.88 ± 5.43	11.15 ± 4.99
* Biceps skinfold thickness (mm)*	3.48 ± 0.71	3.78 ± 0.69
* Triceps skinfold thickness (mm)*	4.23 ± 0.92	4.89 ± 0.92
* Subscapular skinfold thickness (mm)*	4.04 ± 0.84	4.79 ± 0.89
* Sum of skinfold thickness (mm)*	11.74 ± 2.22	13.47 ± 2.07
* EPDS score of mother (post-natal)*	14.24 ± 10.58	10.98 ± 11.00
Mother’s age at baseline (years)		
* < 22*	28 (25.7)	177 (34.2)
* 22 – 25*	47 (43.1)	199 (38.5)
* 26 – 30*	28 (25.7)	110 (21.3)
* 31 – 35*	4 (3.7)	27 (5.2)
* > 35*	2 (1.8)	4 (0.8)
Religion		
* Hinduism*	54 (49.5)	215 (41.6)
* Islam*	50 (45.9)	288 (55.7)
* Christianity*	5 (4.6)	14 (2.7)
Occupation		
* Unemployed*	97 (89.0)	483 (93.4)
* Unskilled*	7 (6.4)	23 (4.4)
* Semi-skilled and skilled*	2 (1.8)	11 (2.2)
Husband’s occupation		
* Unemployed*	2 (1.8)	1 (0.2)
* Unskilled*	55 (50.5)	264 (51.1)
* Semi-skilled*	33 (30.3)	136 (26.3)
* Skilled*	18 (16.5)	94 (18.2)
* Clerical/Semi-professional*	1 (0.9)	22 (4.3)
Kuppuswamy scale		
* Upper*	0	4 (0.8)
* Upper middle*	103 (94.5)	466 (90.1)
* Lower middle*	5 (4.6)	44 (8.5)
* Upper lower*	1 (0.9)	3 (0.6)
Gravida		
* 1*	33 (30.3)	191 (36.9)
* 2*	56 (51.4)	206 (39.8)
* 3*	16 (14.7)	92 (17.8)
* More than 3*	4 (3.7)	28 (5.5)
Parity		
* 0*	35 (32.1)	227 (43.9)
* 1*	68 (62.4)	232 (44.9)
* 2 or more*	6 (5.5)	58 (11.2)
EPDS score (>11) at delivery	23 (21.1)	75 (14.5)
Gender of baby		
* Female*	50 (45.9)	276 (53.4)
* Male*	59 (54.1)	241 (46.6)

Values are presented as mean ± standard deviation or n (%); SGA: small for gestational age; AGA: appropriate for gestational age; EPDS: Edinburgh Postnatal Depression Scale; BMI: body mass index

No major variation was found between the mean and standard deviation for age, gravida, parity and abortion status of mothers with relation to SGA and AGA category. A higher proportion of SGA was found in male babies compared to female babies. Mothers who delivered SGA babies had greater mean EPDS scores during pregnancy (6.27 *vs* 5.73%) and at the time of delivery (21.1 *vs* 14.5%) compared to the mothers who delivered AGA babies. Among the mothers who delivered SGA babies, a majority (68.8%) were younger (under 25 years) and the SGA proportion decreased with the increase in age. Hindus had a higher proportion of delivering SGA babies (49.5%) followed by Muslims (45.9%) and Christians (4.6%) ( Table 3). Education of the partners with higher than high school level had a lesser chance of delivering SGA babies compared to their counterparts.

Adjusted odds ratio (OR) and 95% confidence interval (CI) for EPDS cut off 11, 12, 13 and SGA is presented in Table 4. The EPDS score as a continuous predictor did not show statistically significant association with SGA. A significant association was found between EPDS 11 cutoff and SGA. Women with EPDS scores of above 11 had a twice as high odds of giving birth to a child who would be SGA (Adjusted OR = 2.03; 95% CI = 1.12 - 3.70) compared to the women with EPDS scores of 11 and below. The EPDS 12 (Adjusted OR = 1.96; 95% CI = 1.04 – 3.69) and EPDS 13 (Adjusted OR = 2.42; 95% CI = 1.24 – 4.70) cut-off categories also proved to be a risk factor for SGA with significant p-value (0.0006 and 0.0003), and the individuals with more than 13 EPDS score is found to have the highest odds of SGA

**Table 4.** Association between maternal depressive symptoms during pregnancy and SGA.

*EPDS score*	*Adjusted OR (95% CI)* *for SGA*	*p-value (EPDS* *score in the model)*	*p-value* *(Model)*
EPDS score(Continuous)	1.026 (0.99 – 1.07)	0.212	<0.001
EPDS 11	2.03 (1.12 – 3.70)	0.020	0.001
EPDS 12	1.96 (1.04 – 3.69)	0.037	0.001
EPDS 13	2.42 (1.24 – 4.70)	0.009	<0.001

*SGA: small for gestational age; EPDS: Edinburgh Postnatal Depression Scale*. Adjusted for maternal age, religion, consanguineous marriage, respondent and husband’s education, occupation and income, gravida, parity, anaemia, husband’s current tobacco and alcohol intake and respondent’s sum of skinfold thickness. EPDS categories are defined as follows: EPDS score – continuous variable. **EPDS 11** – EPDS score of either more than 11 or 11 and below. **EPDS 12** – EPDS score of either more than 12 or 12 and below. **EPDS 13** – EPDS score of either more than 13 or 13 and below

## Discussion

Using a longitudinal study, we found that a relationship may exist between the symptoms of mental distress in pregnant women and SGA babies. Using a validated EPDS questionnaire, appropriate for the India populace, we were able to capture scores from 654 expectant mothers during and post-pregnancy. We also found that the prevalence of depressive symptoms was relatively high (16.5%; n=108/654). This was higher compared to our previous study using the Kessler-10 scale (prevalence of 8.7%) across Bangalore ^34^, and is comparable to other Asian countries (20%) and LMICs (15.6%) ^35, 36^.

Further, more salient findings from our analysis showed that pregnant women with depressive symptoms in the second trimester exhibited an increased likelihood of giving birth to SGA infants when assessed using a cut-off value of 11 or above of the EPDS. This association was observed after adjusting for possible confounders: maternal age, religion, consanguineous marriage, respondent and husband's education, occupation, and income, gravida, parity, anaemia, husband's current tobacco and alcohol consumption, and respondent's sum of skinfold thickness. A significant association between scores of 11 or above and SGA were noted (p≤0.005) that were further corroborated with OR - values, while lower EPDS scores were not significantly associated. We believe that mental health problems faced by pregnant women may not be simply and completely measured by EPDS alone, as the perception of stressors may vary and there may be varying levels of buffer mechanisms ^37, 38^. Thus it is essential to explore further these findings based on perception, coping, and interpersonal attitudes ^33, 39, 40^.

Our findings are in concurrence with evidence from other South Asian countries such as Bangladesh ^16, 41, 42^, while the results from high-income countries and sub-Saharan Africa were mostly negative ^43– 45^. The different geographical variations of this association need further exploration. Also, if proven, this understanding of the life-course perspective of the mental health of women in India may help in reducing the prevalence of LBW ^46, 47^.

Earlier studies have shown maternal nutrition to be an important predictor of LBW ^48^. In our study, after adjusting for anaemia, the results from our study suggest that maternal antepartum depression might act independently in causing LBW. While the largest proportion of LBW in India results from poor maternal nutritional status ^45^, there are possibilities that antepartum depression may add to the significant burden of LBW. Evidence from neighbouring countries as Pakistan and Bangladesh supports this finding ^41, 42^. Further proof/evidence that delineates causative pathways leading to LBW and its interactions will provide a unique, compelling opportunity to inform the development of specific preventive interventions for childhood malnutrition. Since LBW is multifactorial in origin and can lead to childhood obesity and its complications, our results indicate psychosocial environment as a potential, contextually relevant risk factor for LBW.

There is a need for establishing the causal association, after which the policymakers can prioritize screening pregnant women for mental health problems. The governments can modify and or/incorporate mental health screening within the existing provisions of the national health mission.

In summary, we were successful in using a simple screening method at the primary care level for screening depression in the antenatal population. Healthcare workers at primary health care levels can thus efficiently screen pregnant women for depression and refer those in need of further care.

There are three potential explanations for the association of antenatal depression and SGA. One, antenatal depression might result in dysregulation of the hypothalamic-pituitary-adrenocortical axis, thereby releasing stress hormones. For example, cortisol levels might mediate this association ^49^, possibly resulting in decreased blood flow to the placenta and consequent restriction of oxygen and nutrients to the fetus leading to intrauterine growth retardation ^50– 54^. To explore this possibility further, mediation mechanisms by cortisol and other catecholamines prospectively is necessary. Two, it is possible that the antenatal depression interacts with other maternal antecedents, such as maternal undernutrition, poor access to healthcare facilities, smoking, alcohol and substance abuse, which are independent known risk factors of LBW ^55^. Such an association may be generally seen in women of disadvantaged social groups. Therefore poverty might confound the association between mental health and LBW. Although we have adjusted for income, there might be a possibility of residual confounding distorting the association. Thirdly, pre-conception depression and mental health status have also been showed to be associated with low birth weight ^56^

### Strengths and limitations

There are various strengths of our study: First, our study is a birth cohort with real-time data quality monitoring. Second, our prospective examination of antenatal depression with SGA is carried out in a sufficiently large study sample. Third, we were able to adjust for several potential confounders; fourth, have also demonstrated the usefulness of the 10-item EPDS screening tool in screening for antenatal depression that can be used even at primary care level. Further, there were few limitations: first, since our study is not immune to the source of systematic error similar to all other observational studies, we are not providing any causal inference regarding the association between EPDS and SGA. Second, we have not recorded pre-pregnancy BMI. Third, we did not assess violence which is a considerable risk factor; and finally, we have not evaluated anxiety as part of the screening and it might be a limitation given that anxiety and depression are known to be co-morbid ^57, 58^.

## Conclusion

Our findings indicate that maternal distress due to depression can lead to the birth of SGA babies. There is a need to universally screen women for depression during pregnancy. The causal links and mediation by other factors have to be delineated before policymakers can consider prioritizing screening and care for mental health, especially in the women belonging to vulnerable or lower socioeconomic backgrounds.

## Ethics and consent

The study was reviewed and approved by the institutional ethical review board at the Bangalore campus of IIPH-H (Ref No: IIPHHB/TRCIEC/091/2015 Dated 13/11/2015).

Written informed consent has been obtained from all the enrolled participants of the study.

## Data availability

Dataset 1: Raw data for the study ‘Small for gestational age babies and depressive symptoms of mothers during pregnancy: Results from a birth cohort in India’ available on OSF: http://doi.org/10.17605/OSF.IO/BV8F6 ^59^.

Data are available under the terms of the Creative Commons Zero "No rights reserved" data waiver (CC0 1.0 Public domain dedication).

### Extended data

Supplementary File 1. SGA and EPDS_Supplementary tables.

 The file contains tables on:

1) Association between antenatal depression and SGA (adjusted for interaction effect of the cofounders), and

2) Association between EPDS score of cut off 10 and SGA (adjusted for the confounders)

Available from figshare: DOI: 10.6084/m9.figshare.11771226
