# Supplementary material for: Small for gestational age babies and depressive symptoms of mothers during pregnancy: Results from a birth cohort in India
Source: Wellcome Open Res. 2020 Feb 6;3:76. Originally published 2018 Jun 21. [Version 3] doi: 10.12688/wellcomeopenres.14618.3 (PMC6892423; doi:10.12688/wellcomeopenres.14618.3)
Supplement: Peer review history

# Peer review: Reviewer response for version 3

**DOI:** 10.21956/wellcomeopenres.17232.r37835

**Published:** 2020-03-10

**Authors:** Jodie G. Katon

**Affiliations:** 1 Health Services Research and Development (HSR&D) Center of Innovation for Veteran-Centered and Value-Driven Care, US Department of Veterans Affairs (VA) Puget Sound Health Care System, Seattle, WA, USA

I have read the latest revision of the manuscript and found it very responsive to past reviewer comments. At this time I recommend for indexing.

I confirm that I have read this submission and believe that I have an appropriate level of expertise to confirm that it is of an acceptable scientific standard.

# Peer review: Reviewer response for version 3

**DOI:** 10.21956/wellcomeopenres.17232.r37837

**Published:** 2020-03-10

**Authors:** Jonathan Y. Huang

**Affiliations:** 1 Singapore Institute for Clinical Sciences (SICS), Agency for Science, Technology and Research (A*STAR), Singapore, Singapore

Here I quote from my previous review some points that I think could use additional clarification. I hope they are helpful in further developing this work.

 “First, the authors could better consider the association between EPDS as a continuous score, including the potential for non-linearity as they suggest may occur and present this as a main not supplemental finding (as it will also address their secondary aim).”

- *Thank you for presenting the continuous results. Perhaps one thing to check, is whether there are enough subjects and events at lower EPDS scores. Since your discussion suggests that 11 is a critical threshold and potentially useful for screening (though again, prediction modelling should be handled separately), it would make sense, at minimum to evaluate at other scores where there are sufficient sample sizes, e.g. 10 (move from the supplement), 9, 8 (thresholds used in other settings), so that we get a sense of the distribution of SGA across the continuum and whether there is any indication of “dose-response” or whether those thresholds are not as strongly associated. A cross-tab by EPDS score, would be very useful descriptive information.*

“Second, while it is heartening that authors specified a priori covariates, the high likelihood of residual confounding should direct them to consider other additional measures at their disposal in this cohort study including maternal BMI, blood pressure, gestational diabetes or dysgylcemia (which, given its etiology, will precede their collection of EPDS in mid/late pregnancy), and maternal medical history, especially, if available, medication.”

- *I appreciate the addition of some measure of maternal adiposity (skinfold thickness) as a covariate, however I’m not sure why BMI could not be used/included as the authors spend an entire paragraph describing the protocol for “Adult Anthropometry” which includes standing height and weight. I am also still puzzled, given the context (MAASTHI) is an adiposity and metabolism cohort, why other pregnancy conditions and medical history were not included in models. I should note here that the methods now report different covariates than the discussion and Table 4 caption, so they need to be harmonized.*
- *Also, it is not clear why concern with “multi-collinearity” would require removal of all measures of socioeconomic status from the model. Surely, at minimum, maternal education and household income could be retained? There is often important and critical variation between these factors relevant to maternal depressive symptoms (e.g. from mismatch between education and income). From Table 1, it is interesting to note, for example, that there are higher proportions of both middle school and graduate+ educated mothers among EDPS > 11 (vs <11), suggestive of a mixed SES group.*

“Third, they had hypothesized that associations may differ based on socioeconomic status, yet they did not evaluate any interactions by such factors. If any were specified a priori, they would be interesting to present (even if “not significant”).”

- *Author have not yet addressed potential interaction with SES despite it being alluded to in the Introduction as an important source of variation and therefore contribution to the literature regarding the local context. For example, consistent or different associations across SES groups would provide some insight as to why results from Bangalore are different from other contexts. *

“Fourth, the cohort has commendably extensive measures of anthropometrics. It is surprising, given findings they have cited (e.g. Broekman, *et al*. (2014) ^4^) that they did not investigate associations with parameters other than SGA.”

- *In addition, in light of all the effort put into collecting the other anthropometric measures and reporting of measurement methodology in the manuscript, I’m also not clear why associations between EPDS and those other anthropometric measures were not reported given, as above, past findings with respect to head circumference. These results should be provided as well to avoid the impression of selective reporting. In contrast, Table 3 which reports anthropometry (and other measures) stratified by SGA/AGA are not as informative to your main hypothesis.*

 “The flow chart is missing some information…Since mothers were recruited in 2 ^nd^ and 3 ^rd^ trimester, it is surprising that so large a fraction would be lost to follow-up. Since depression and fetal health may both be related to loss-to-follow-up there is a possibility that any associations found in your final sample may be subject to selection bias. At the very least, baseline characteristics of recruited participants should be reported, stratified by whether they are retained in your final sample.”

- *The authors still need to address why there is such a striking loss (794/1557 = 51% loss) from late pregnancy depression assessment (N = 1557) to live births (N = 763) when only 9 are accounted for from either twin or still birth. As noted before both maternal symptoms and fetal health may be related to loss-to-follow-up, so it is important to note why so few were followed up at birth. At minimum, it should be stated, e.g. how many subjects could not be traced or refused to participate vs. how many had pregnancy loss. Adding baseline data for those lost to follow-up in a supplement would also be helpful.*

“In Table 4, it is not entirely clear which tests the two columns of p-values refer to. Assuming “EPDS score in the model” refer to the p-value for the specific coefficient and “model” refers to the p-value for the goodness-of-fit test including all covariate, this should be clarified in the caption text. Undue precision in the estimates is also discouraged. For example, 2 decimal places for odds ratios and 3 for p-values are probably sufficient.”

- *I’m afraid I’m still confused about this, can the captions be clarified with what tests the p-values refer to?*
- *Finally, thank you for addressing the formatting issues for the Tables and presenting the EPDS stratified Table 1. They lead to interesting observations (e.g. the difference in maternal education distribution noted above). Table 2 does not present any mean +/- SD, so it can probably be removed from the caption to avoid confusion. Again, I don’t know that Table 3 is necessary or a main finding.*

I confirm that I have read this submission and believe that I have an appropriate level of expertise to confirm that it is of an acceptable scientific standard, however I have significant reservations, as outlined above.

# Peer review: Reviewer response for version 3

**DOI:** 10.21956/wellcomeopenres.17232.r37834

**Published:** 2020-02-14

**Authors:** Howard Cabral

**Affiliations:** 1 Department of Biostatistics, Boston University School of Public Health, Boston, MA, USA

This paper examines the association of maternal depressive symptoms during pregnancy and small for gestation age delivery in a birth cohort in India from April 2016 to October 2017. The paper is generally well written and the tables and figures are well done and informative. The authors have addressed the remaining points from the prior review.

I confirm that I have read this submission and believe that I have an appropriate level of expertise to confirm that it is of an acceptable scientific standard.

# Peer review: Reviewer response for version 2

**DOI:** 10.21956/wellcomeopenres.16498.r37240

**Published:** 2019-12-02

**Authors:** Jodie G. Katon

**Affiliations:** 1 Health Services Research and Development (HSR&D) Center of Innovation for Veteran-Centered and Value-Driven Care, US Department of Veterans Affairs (VA) Puget Sound Health Care System, Seattle, WA, USA

This manuscript explores the association of maternal depression during pregnancy with infant birth size.  The primary outcome was small for gestational age (SGA) defined based on population based birth weight percentiles.  Notably the investigators also collected a broad range of infant anthropometric measures.  A secondary aim of the study was to determine the utility of depression symptoms as measured by the Edinburgh Perinatal Depression Scale (EPDS) for predicting SGA.  As noted by the authors despite the high prevalence of both SGA and perinatal depression in low and middle income countries the majority of the research on the associations of these outcomes has been in high income, Western countries (though there are notable exceptions).  The authors further note that given the growing concern regarding diseases such as diabetes and cardiovascular disease in low and middle income countries and the potential links with low birth weight via the “thrifty phenotype” hypothesis that there are important long term population health implications for failing to understand and address the role of perinatal depression in fetal growth restriction.  The cohort described in this analysis provides a potentially powerful data set to begin to address these questions.

 Given the number of infant anthropometric measures and questions regarding the cut-off of the EPDS score raised by other reviewers I suggest the following:

1. Focus the manuscript on associations of EPDS and infant anthropometry (beyond just SGA).
2. Remove the analysis on the predictive capabilities of EPDS for SGA, as this could be further developed in to a secondary manuscript.
3. Consider not just dichotomizing EPDS but consider looking at it as a continuous predictor as well as a non-linear predictor.
4. Large for gestational age (LGA) is only mentioned briefly in the methods and results, but I find it interesting that LGA is also higher among those with symptoms of depression.  Focusing the manuscript more on the associations EPDS with infant anthropometry might enable further discussion of this finding.  If you don’t explore further I would consider removing as it is otherwise distracting and difficult to reconcile with the primary findings.

Minor points regarding organization and data presentation:

1. Tables include means and categories for continuous measures (e.g. age), either chose one (I would recommend categories) or if both are retained group them together in the tables (e.g. mean age followed by the age categories).
2. Move the Total column to be the first column in Table 1.  This will enable the authors to first describe overall cohort characteristics and then to focus on descriptive comparisons based on EPDS score.

I confirm that I have read this submission and believe that I have an appropriate level of expertise to confirm that it is of an acceptable scientific standard, however I have significant reservations, as outlined above.

# Response

**Published:** 2020-01-30

**Authors:** Giridhara R Babu

**Affiliations:** Public Health Foundation of India, India

Focus the manuscript on associations of EPDS and infant anthropometry (beyond just SGA).

**Response:** Many thanks for the suggestion, however, at this point we would like to share that we have decided to present the associations of EPDS and infant anthropometry as a separate paper. Hence adding it now, is beyond the scope of the paper. 

Remove the analysis on the predictive capabilities of EPDS for SGA, as this could be further developed into a secondary manuscript. 

**Response:** Many thanks for this important suggestion as well. We have made sure to remove all the analysis on the predictive capabilities of EPDS for SGA, and as suggested planned another paper based on the same.

Consider not just dichotomizing EPDS but consider looking at it as a continuous predictor as well as a non-linear predictor.

**Response: **Thank you for suggestion. We have included this in Table.4.

Large for gestational age (LGA) is only mentioned briefly in the methods and results, but I find it interesting that LGA is also higher among those with symptoms of depression.  Focusing the manuscript more on the associations EPDS with infant anthropometry might enable further discussion of this finding.  If you don’t explore further I would consider removing as it is otherwise distracting and difficult to reconcile with the primary findings. 

**Response:** Many thanks for this important suggestion. We duly accept the suggestion and have made sure to remove all the analysis LGA. 

Minor points regarding organization and data presentation:

Tables include means and categories for continuous measures (e.g. age), either chose one (I would recommend categories) or if both are retained group them together in the tables (e.g. mean age followed by the age categories). 

**Response: **Thank you for the suggestion. Changes are made accordingly.

Move the Total column to be the first column in Table 1.  This will enable the authors to first describe overall cohort characteristics and then to focus on descriptive comparisons based on EPDS score. 

**Response**: Changes are made in the table as per suggestion.

# Peer review: Reviewer response for version 2

**DOI:** 10.21956/wellcomeopenres.16498.r35986

**Published:** 2019-08-06

**Authors:** Jonathan Y. Huang

**Affiliations:** 1 Singapore Institute for Clinical Sciences (SICS), Agency for Science, Technology and Research (A*STAR), Singapore, Singapore

This study seeks to demonstrate associations between reported maternal depression symptoms and risk for small-for-gestational-age (SGA) birth. The motivation is that SGA is related to substantial subsequent morbidity, the role of maternal depression in SGA is underexplored in the Indian context, and implicitly, this relationship in India may differ from more commonly studied countries. The authors should be commended for the strengths of their study particularly the live ascertainment of EPDS and the extensive anthropometry collected in neonates.  

 In general, this study supports a large, if inconsistent body, of past findings and meta-analyses (Szegda *et al*. (2013) ^1^, Eastwood *et al*. (2017) ^2^) that support an association between depressive symptoms as measured by the EPDS and adverse birth outcomes such as LBW, PTB, or in this case, SGA. On the other hand, the study does not move towards filling the noted gaps in current understanding of the causal nature of maternal depressive symptoms, including 1) whether associations merely reflect the importance of pre-conception depressive symptoms, 2) whether key covariates such as medication usage and medical history (Zhao, *et al*. (2018) ^3^) can be accounted for, and 3) as mentioned by other reviewers, whether associations remain across subclinical scores. Nor does it address some of the local needs they refer to in their introduction.

 Within the constraints of their current design, the authors could potentially improve upon the literature and their analyses in a few key ways:

 First, the authors could better consider the association between EPDS as a continuous score, including the potential for non-linearity as they suggest may occur and present this as a main not supplemental finding (as it will also address their secondary aim).

 Second, while it is heartening that authors specified a priori covariates, the high likelihood of residual confounding should direct them to consider other additional measures at their disposal in this cohort study including maternal BMI, blood pressure, gestational diabetes or dysgylcemia (which, given its etiology, will precede their collection of EPDS in mid/late pregnancy), and maternal medical history, especially, if available, medication.

 Third, they had hypothesized that associations may differ based on socioeconomic status, yet they did not evaluate any interactions by such factors. If any were specified a priori, they would be interesting to present (even if “not significant”).

 Fourth, the cohort has commendably extensive measures of anthropometrics. It is surprising, given findings they have cited (e.g. Broekman, *et al*. (2014) ^4^) that they did not investigate associations with parameters other than SGA.

 Finally, given the possibility that depressive symptoms may reflect pre-conception depression and the likelihood of residual confounding, discussion of “effects” or benefit of intervention during pregnancy should be minimized. The authors should be commended for mentioning this in their conclusions, however the extent of residual confounding and selection bias (see “major point” below) is not given sufficient weight in their “three potential explanations” paragraph.

 A secondary aim appeared to be exploring whether a threshold for EPDS could be used for screening on the basis of SGA risk. Risk prediction models may be valid even with absent causal interpretations, however, the development of screening tools require more inputs that simplify the performance of a particular set of predictors, in particular giving due consideration for the contexts for decision making. Notably, an AUROC of 0.76 alone is not sufficient information to determine if the EPDS is a useful as a diagnostic or screening tool for SGA. Other characteristics such as positive predictive value (“precision”) and accuracy need to be presented, taking into account decision support considerations such as the cost/penalty of false positives, given for example, the 15% false positive fraction for depression at EPDS >= 13 the authors cited. Again, EPDS scores need not be dichotomized as a predictor of SGA and may be assessed continuously and non-linearly. Notably, given three dichotomous cutpoints and looking only at goodness-of-fit and AUROC, the authors have not well investigated whether “peak adversities of SGA” occur at a score of 11. A continuous measure would be more informative in this regard. Finally, one key missing piece of information from the current analyses is the degree to which addition of EPDS improves the classification of SGA over models without the score.  

 Major points: The flow chart is missing some information. The count and percentage of live births do not seem to line up: 763 is 49% of 1557 recruited. After excluding the 5 twins and 4 still births, there are still 785 (50%) not accounted for. It is important to know the disposition of this group, what % withdrew, lost contact, miscarried, etc? Since mothers were recruited in 2 ^nd^ and 3 ^rd^ trimester, it is surprising that so large a fraction would be lost to follow-up. Since depression and fetal health may both be related to loss-to-follow-up there is a possibility that any associations found in your final sample may be subject to selection bias. At the very least, baseline characteristics of recruited participants should be reported, stratified by whether they are retained in your final sample. More rigorously, they could see the sensitivity of their findings using regressions that are weighted for an inverse probability of selection into the final sample. Such a weight could be created by using logistic regression with the outcome being an indicator of censorship (1 if lost to follow-up, 0 if observed) predicted by all available covariates. While this will not fully correct for selection based on unobserved characteristics, it can help demonstrate the direction of the bias.    

 Minor points: There is not a strong need to report both mean and counts for the same variables (e.g. age, gravidity and parity) in the model. For gravidity and parity, cell counts can provide more information if they are exhaustive. In contrast, for age, the wide categories including truncation at 22 years are somewhat arbitrary when the mean (or likely median) suggest, for example, there is little difference in age distributions by EPDS threshold.

 In the Results, if the goal is to describe differences in characteristics between mothers below/above the EPDS threshold (i.e. in line with Table 1), authors should provide both sets of results so readers can make the explicit comparison. For example, if authors wish to report that “60% of husbands [of women with EPDS > 11] were consuming tobacco” it would be helpful to present that only 42% of husbands of EPDS <= 11 women. Why weren’t abortions reported in Table 1?

 In Table 4, it is not entirely clear which tests the two columns of p-values refer to. Assuming “EPDS score in the model” refer to the p-value for the specific coefficient and “model” refers to the p-value for the goodness-of-fit test including all covariate, this should be clarified in the caption text. Undue precision in the estimates is also discouraged. For example, 2 decimal places for odds ratios and 3 for p-values are probably sufficient.

 If available, consideration of physical activity and plasma vitamin D may be beneficial.

I confirm that I have read this submission and believe that I have an appropriate level of expertise to state that I do not consider it to be of an acceptable scientific standard, for reasons outlined above.

## References

[1] : Depression during pregnancy: a risk factor for adverse neonatal outcomes? A critical review of the literature. *J Matern Fetal Neonatal Med*.2014;27(9) : 10.3109/14767058.2013.845157 960-7 10.3109/14767058.2013.845157 24044422PMC4812822

[2] : The Impact of Antenatal Depression on Perinatal Outcomes in Australian Women. *PLoS One*.2017;12(1) : 10.1371/journal.pone.0169907 e0169907 10.1371/journal.pone.0169907 28095461PMC5241141

[3] : A meta-analysis of selective serotonin reuptake inhibitors (SSRIs) use during prenatal depression and risk of low birth weight and small for gestational age. *Journal of Affective Disorders*.2018;241: 10.1016/j.jad.2018.08.061 563-570 10.1016/j.jad.2018.08.061 30153640

[4] : The influence of anxiety and depressive symptoms during pregnancy on birth size. *Paediatr Perinat Epidemiol*.2014;28(2) : 10.1111/ppe.12096 116-26 10.1111/ppe.12096 24266599

# Response

**Published:** 2020-01-30

**Authors:** Giridhara R Babu

**Affiliations:** Public Health Foundation of India, India

First, the authors could better consider the association between EPDS as a continuous score, including the potential for non-linearity as they suggest may occur and present this as a main not supplemental finding (as it will also address their secondary aim). 

**Response**: Thank you for the suggestion. We have included the results of association between EPDS as a continuous score and SGA. (Table-4)

Second, while it is heartening that authors specified a priori covariates, the high likelihood of residual confounding should direct them to consider other additional measures at their disposal in this cohort study including maternal BMI, blood pressure, gestational diabetes or dysgylcemia (which, given its etiology, will precede their collection of EPDS in mid/late pregnancy), and maternal medical history, especially, if available, medication. 

**Response**: We do not have maternal pre-pregnancy BMI in this study but have adjusted for maternal skinfold thickness which is a better marker for adiposity during pregnancy considering our cohort participants were recruited into study at different stages of gestation (14 to 36 weeks).

Third, they had hypothesized that associations may differ based on socioeconomic status, yet they did not evaluate any interactions by such factors. If any were specified a priori, they would be interesting to present (even if “not significant”). 

**Response**: We have considered respondent’s as well as husband’s income in the model. Since, income is a criterion for socio economic status, in order to avoid the problem of multi-collinearity we excluded socioeconomic status from the model.

Fourth, the cohort has commendably extensive measures of anthropometrics. It is surprising, given findings they have cited (e.g. Broekman, et al. (2014)4) that they did not investigate associations with parameters other than SGA.

**Response**: Thank you for mentioning it. Since the aim of this study is to examine if the relation between Edinburgh Postnatal Depression Scale (EPDS) score and SGA, we focused on SGA as outcome. 

Finally, given the possibility that depressive symptoms may reflect pre-conception depression and the likelihood of residual confounding, discussion of “effects” or benefit of intervention during pregnancy should be minimized. The authors should be commended for mentioning this in their conclusions, however the extent of residual confounding and selection bias (see “major point” below) is not given sufficient weight in their “three potential explanations” paragraph. 

**Response**: Thank you, we have now included this in the manuscript “Thirdly, pre-conception depression and mental health status have also been showed to be associated with low birth weight

A secondary aim appeared to be exploring whether a threshold for EPDS could be used for screening on the basis of SGA risk. Risk prediction models may be valid even with absent causal interpretations, however, the development of screening tools require more inputs that simplify the performance of a particular set of predictors, in particular giving due consideration for the contexts for decision making. Notably, an AUROC of 0.76 alone is not sufficient information to determine if the EPDS is a useful as a diagnostic or screening tool for SGA. Other characteristics such as positive predictive value (“precision”) and accuracy need to be presented, taking into account decision support considerations such as the cost/penalty of false positives, given for example, the 15% false positive fraction for depression at EPDS >= 13 the authors cited. Again, EPDS scores need not be dichotomized as a predictor of SGA and may be assessed continuously and non-linearly. Notably, given three dichotomous cut points and looking only at goodness-of-fit and AUROC, the authors have not well investigated whether “peak adversities of SGA” occur at a score of 11. A continuous measure would be more informative in this regard. Finally, one key missing piece of information from the current analyses is the degree to which addition of EPDS improves the classification of SGA over models without the score.  

**Response**: Thank you for the suggestions. We have removed the analysis on the predictive capabilities of EPDS for SGA. We have included the analysis results using EPDS score as a continuous predictor.

Major points: The flow chart is missing some information. The count and percentage of live births do not seem to line up: 763 is 49% of 1557 recruited. After excluding the 5 twins and 4 still births, there are still 785 (50%) not accounted for. It is important to know the disposition of this group, what % withdrew, lost contact, miscarried, etc? Since mothers were recruited in 2nd and 3rd trimester, it is surprising that so large a fraction would be lost to follow-up. Since depression and fetal health may both be related to loss-to-follow-up there is a possibility that any associations found in your final sample may be subject to selection bias. At the very least, baseline characteristics of recruited participants should be reported, stratified by whether they are retained in your final sample. 

**Response**: We have considered woman accessed for depressive symptom as sample population for the study. This excludes twins and still birth. Also, since cohort in ongoing, all the women have not delivered when the current study was conducted. Only 50% of mothers delivered, out of those who were recruited into the study during the time of current study. We have considered so far delivered women and whose followup had been completed on time. For the analysis, we have considered 654 respondents whose EPDS score had been assessed during pregnancy and who had delivered and followup completed on time. We have updated the figure.

More rigorously, they could see the sensitivity of their findings using regressions that are weighted for an inverse probability of selection into the final sample. Such a weight could be created by using logistic regression with the outcome being an indicator of censorship (1 if lost to follow-up, 0 if observed) predicted by all available covariates. While this will not fully correct for selection based on unobserved characteristics, it can help demonstrate the direction of the bias.  

**Response**: We have removed the findings.

Minor points: There is not a strong need to report both mean and counts for the same variables (e.g. age, gravidity and parity) in the model. For gravidity and parity, cell counts can provide more information if they are exhaustive. In contrast, for age, the wide categories including truncation at 22 years are somewhat arbitrary when the mean (or likely median) suggest, for example, there is little difference in age distributions by EPDS threshold. 

**Response**: Thank you, we have now modified the table and retained the cell count for Gravidity and parity and have mentioned mean age.

In the Results, if the goal is to describe differences in characteristics between mothers below/above the EPDS threshold (i.e. in line with Table 1), authors should provide both sets of results so readers can make the explicit comparison. For example, if authors wish to report that “60% of husbands [of women with EPDS > 11] were consuming tobacco” it would be helpful to present that only 42% of husbands of EPDS <= 11 women. Why weren’t abortions reported in Table 1? 

**Response**: Since gravida and parity are included, we excluded still birth and abortion to concise the information.

In Table 4, it is not entirely clear which tests the two columns of p-values refer to. Assuming “EPDS score in the model” refer to the p-value for the specific coefficient and “model” refers to the p-value for the goodness-of-fit test including all covariate, this should be clarified in the caption text. Undue precision in the estimates is also discouraged. For example, 2 decimal places for odds ratios and 3 for p-values are probably sufficient. 

**Response**: Thank you for your suggestion. Changes are made accordingly.

If available, consideration of physical activity and plasma vitamin D may be beneficial.

**Response**: Thank you for the suggestion, however, we have not yet assessed the vitamin D levels in the stored samples. We will assess these in a future manuscript

# Peer review: Reviewer response for version 2

**DOI:** 10.21956/wellcomeopenres.16498.r35333

**Published:** 2019-04-24

**Authors:** Howard Cabral

**Affiliations:** 1 Department of Biostatistics, Boston University School of Public Health, Boston, MA, USA

This paper examines the association of maternal depressive symptoms during pregnancy and small for gestation age delivery in a birth cohort in India from April 2016 to October 2017. The paper is generally well written and the tables and figures are well done and informative.

 There are several points raised in the prior review that have not been addressed in the revised text.

 Among the important confounding variables not included in the analysis would indeed be exposure to violence, a factor that is often not included in similar studies, though it clearly should be if available given that depressive symptomatology is the primary independent variable here. Checking the effects of applying different cutoffs to the Edinburgh (EPDS) score is helpful from a clinical standpoint, though the intent of developing a score is to be able to identify risk that is subclinical. Hence, analyses that use the EPDS score as continuous would also be informative. Women with scores less than a cutoff are indeed not “without mental depressive symptoms”. The authors note that they have performed analyses using the continuous score but this is not apparent in the Methods or Results but in a Supplemental file. If this is the accepted approach of the publishing platform, this is fine but a link to this information of results should be included in the main text also.

 The authors state that additional statistical analyses checked for effect modification (interaction) with depressive symptoms for salient variables on intrauterine growth. The methods and results of these models are not shown in the main text. Are these included in the Supplemental File also? If so, the recommendation above applies here also. If the interactions were found to be statistically and clinically significant, then showing the main effects only model as the primary set of results is inappropriate.

 As noted above with respect to exposure to violence, very important confounders are not included in the statistical models that could alter the estimation of the effect of depressive symptoms on intrauterine growth. These would include maternal pre-pregnancy weight or BMI, as well as maternal health habits that have been shown to have associations with depressive symptoms, including maternal substance use of various kinds and the quality of prenatal care. A list of the most important confounders that were not examined in this study should be included in the limitations. 

 The English grammar in the text should be thoroughly re-checked.

I confirm that I have read this submission and believe that I have an appropriate level of expertise to confirm that it is of an acceptable scientific standard, however I have significant reservations, as outlined above.

# Response

**Published:** 2020-01-30

**Authors:** Giridhara R Babu

**Affiliations:** Public Health Foundation of India, India

- Among the important confounding variables not included in the analysis would indeed be exposure to violence, a factor that is often not included in similar studies, though it clearly should be if available given that depressive symptomatology is the primary independent variable here. Checking the effects of applying different cutoffs to the Edinburgh (EPDS) score is helpful from a clinical standpoint, though the intent of developing a score is to be able to identify risk that is subclinical. Hence, analyses that use the EPDS score as continuous would also be informative. Women with scores less than a cutoff are indeed not “without mental depressive symptoms”. The authors note that they have performed analyses using the continuous score but this is not apparent in the Methods or Results but in a Supplemental file. If this is the accepted approach of the publishing platform, this is fine but a link to this information of results should be included in the main text also.

**Response**: Thank you for the suggestion. We have included the analysis results using EPDS score as a continuous predictor.

- The authors state that additional statistical analyses checked for effect modification (interaction) with depressive symptoms for salient variables on intrauterine growth. The methods and results of these models are not shown in the main text. Are these included in the Supplemental File also? If so, the recommendation above applies here also. If the interactions were found to be statistically and clinically significant, then showing the main effects only model as the primary set of results is inappropriate.

**Response**: The results of interaction are provided in the supplement table. Interactions were not statistically significant.

- As noted above with respect to exposure to violence, very important confounders are not included in the statistical models that could alter the estimation of the effect of depressive symptoms on intrauterine growth. These would include maternal pre-pregnancy weight or BMI, as well as maternal health habits that have been shown to have associations with depressive symptoms, including maternal substance use of various kinds and the quality of prenatal care. A list of the most important confounders that were not examined in this study should be included in the limitations.

**Response**: Unfortunately, we don’t have the data regarding exposure to violence and pre-pregnancy BMI, this is one of the limitations of our study. We have mentioned it in our manuscript as a limitation of study. Instead of pre-pregnancy BMI, we included maternal adiposity measured through skinfold thickness as a confounder. Maternal substance use is very minimal (less than 1%) in the study sample, we have adjusted for the husband's current tobacco and alcohol consumption as indicator domestic violence.

- The English grammar in the text should be thoroughly re-checked.

**Response**: We have thoroughly revised the grammar of the manuscript.

# Peer review: Reviewer response for version 1

**DOI:** 10.21956/wellcomeopenres.15915.r33919

**Published:** 2018-11-14

**Authors:** Nisreen A. Alwan

**Affiliations:** 1 Academic Unit of Primary Care and Population Sciences, Faculty of Medicine, Southampton General Hospital, University of Southampton, Southampton, UK

This is an observational study which measured maternal depressive symptoms during pregnancy using the Edinburgh Postnatal Depression Scale (EPDS), and examined if this is linked to having a small for gestational age (SGA) birth in the MAASTHI birth cohort in India.

 The stated study aim in the manuscript is to “replicate the association between antepartum depression and SGA in the setting of a public hospital in India”, however the abstract conclusion seems to comment on the validity of using EPDS as a screening tool for antenatal depression. The study does not explicitly state the aim of examining the validity of EPDS as a screening tool. The abstract also reports values for the AUC using different cut-offs of EPDS for the diagnosis of antenatal depression. These values are only in relation to the SGA outcome examined in this study and does not compare EPDS to a ‘gold standard’ or another screening test for antenatal depression. Therefore, it is not accurate to comment of the “usefulness of using 10-item EPDS screening tool” in relation to other outcomes other than SGA, or for use as a screening tool in general.

 The manuscript needs to be clear about this, and if the authors would like to keep the ‘prediction’ element of EPDS in relation to SGA as an outcome, they need to be clear about this in the aims and methods.

 Under the Methods section-Measurement, the authors state that they “aimed to assess the exact EPDS score cut-off value (11,12 or 13) as a better predictor of association between antenatal depression and SGA”. Firstly, this statement needs to move to the aims section at the end of the Introduction section, and also needs to be clearly stated in the abstract. Secondly, this aim is not interchangeable with testing if EDPS is a valid screening tool for antenatal depression in the population the study is trying to generalise results to.

 Under the Statistical Analysis section, it is not clear whether the association with SGA was examined using the continuous EPDS score or the 3 categorical variables based on the cut-off scores of 11, 12 and 13, or both.

 Was maternal body mass index taken into account as a confounder?

 Under the Results section, second paragraph: "among mothers with depressive symptoms….” using what EPDS cut-off? This applies to all the descriptive findings.

 It is strange that the direction of effect is so different between using a cut-off of 11 versus 12 or 13 of the same scale (aOR 2.18 versus 0.46 and 0.41). Please check your categories and what you have assigned as a reference in your models.  

 Last paragraph of the results section, ‘accuracy of EPDS scale’ in relation to what? Are you saying that the strength of association with one outcome (SGA) a measure of accuracy of the screening test? Please clarify. If you are trying to predict the outcome then that is a function of other factors accounted for in the prediction model (if it is adjusted), not just the EPDS cut-off.

I confirm that I have read this submission and believe that I have an appropriate level of expertise to state that I do not consider it to be of an acceptable scientific standard, for reasons outlined above.

# Response

**Published:** 2019-02-12

**Authors:** Giridhara R Babu

**Affiliations:** Public Health Foundation of India, India

**1. The stated study aim in the manuscript is to “replicate the association between antepartum depression and SGA in the setting of a public hospital in India”,** **however** **the abstract conclusion seems to comment on the validity of using EPDS as a screening tool for antenatal depression. The study does not explicitly state the aim of examining the validity of EPDS as a screening tool. The abstract also reports values for the AUC using different cut-offs of EPDS for the diagnosis of antenatal depression. These values are only in relation to the SGA outcome examined in this study and does not compare EPDS to a ‘gold standard’ or another screening test for antenatal depression. Therefore, it is not accurate to comment** **of** **the “usefulness of using 10-item EPDS screening tool” in relation to other outcomes other than SGA, or for use as a screening tool in general.** 

 Thank you for the comments. We have modified the abstract conclusion and result section as per the suggestion.

**2. The manuscript needs to be clear about this, and if the authors would like to keep the ‘prediction’ element of EPDS in relation to SGA as an outcome, they need to be clear about this in the aims and methods.** 

 We have used antenatal depression as the exposure and SGA as an outcome. We have mentioned it clearly in the aims and methods.

**3. Under the Methods section-Measurement, the authors state that they “aimed to assess the exact EPDS score cut-off value (11,12 or 13) as a better predictor of association between antenatal depression and SGA”. Firstly, this statement needs to move to the aims section at the end of the Introduction section, and also needs to be clearly stated in the abstract. Secondly, this aim is not interchangeable with testing if EDPS is a valid screening tool for antenatal depression in the population the study is trying to generalise results** **to** **.** 

 We sincerely thank the reviewer for the comment. The aim of the study is now modified as per the suggestion of the reviewer. We agree with the reviewer that the aim is not interchangeable with testing if EDPS as a valid screening tool for antenatal depression in the population. Clearly, we do not have the intent of doing so. There is no external validity (generalization) without meeting the internal validity. Since our study not immune to the source of systematic error similar to all other observational studies, we are not providing any causal inference regarding the association between EPDS and SGA. We have included this limitation in the revised manuscript.

**4. Under the Statistical Analysis section, it is not clear whether the association with SGA was examined using the continuous EPDS score or the 3 categorical variables based on the cut-off scores of 11, 12 and 13, or both.** 

- The legends of tables contain the categorical classification of EPDS score as per the cut-offs as 11, 12 and 13
- Association with SGA was examined using EPDS score as categorical variable based on the cut off values. We have updated the details in the Statistical Analysis section as well **.(Page 9 Line 6)**

 

**5. Was maternal body mass index taken into account as a confounder?** 

 As we have no data on pre-pregnancy BMI we have not considered the body mass index obtained during different trimester of pregnancy as a confounder, but we have taken sum of skinfold thickness into account. ( 1)

**6. Under the Results section, second paragraph: "among mothers with depressive symptoms….” using what EPDS cut-off? This applies to all the descriptive findings.** 

 Here depressive symptom is defined as EPDS score >11 as we have mentioned in Table 1 and it applies for all descriptive findings. In the present study the cutoff score 13 showed highest OR compared to rest two categories, however, we have shown the descriptive statistics with cutoff of 11 since it is the minimum value at which we got statistically significant results.

**7. It is strange that the direction of effect is so different between using a cut-off of 11 versus 12 or 13 of the same scale (aOR 2.18 versus 0.46 and 0.41). Please check your categories and what you have assigned as a reference in your models. ** 

 We sincerely thank the reviewer for this input. Please note that there was a mistake in coding the variable (EPDS score cut off 11, 12, 13). We recoded the entire data set and have thoroughly checked the entire analysis after redoing it. The resulted OR changes gradually from one cut off category to another. (OR : 2.03 ,1.96, 2.42 respectively)

**8. Last paragraph of the results section, ‘accuracy of EPDS scale’ in relation to what? Are you saying that the strength of association with one outcome (SGA) a measure of** **accuracy** **of the screening test? Please clarify. If you are trying to predict the outcome then that is a function of other factors accounted for in the prediction model (if it is adjusted), not just the EPDS cut-off.** 

 In our study, the use of EPDS score without adjusting for its confounders resulted in very low specificity in predicting SGA. The area under ROC curve using EPDS score alone in predicting SGA was 0.515. EPDS is a screening tool and hence may not fare well as a diagnostic test. However, after adjusting for confounders, the accuracy improved. Therefore, we meant that accuracy in predicting SGA by using EPDS scale improves after accounting for other variables confounders. This section is modified. (Page 18 Line 1)

 1.         Piers L, Soares M, Frandsen S, O'dea K. Indirect estimates of body composition are useful for groups but unreliable in individuals. International journal of obesity. 2000;24(9):1145.

# Peer review: Reviewer response for version 1

**DOI:** 10.21956/wellcomeopenres.15915.r33915

**Published:** 2018-10-08

**Authors:** Howard Cabral

**Affiliations:** 1 Department of Biostatistics, Boston University School of Public Health, Boston, MA, USA

This paper examines the association of maternal depressive symptoms during pregnancy and small for gestation age delivery in a birth cohort in India from April 2016 to October 2017. The paper is generally well written and the tables and figures are well done and informative.

 A number of points of concern, however, can be raised regarding this paper. Among these are points raised in a prior review by Dr. Desai, all of which are very pertinent. The inclusion of fetal loss deliveries would not be appropriate. If these were excluded the sample should be described as one comprised of livebirths only. Also, the inclusion of multiples would render as inappropriate analyses that assume independent observations. Not accounting for potential clustering by clinical site would additionally be inappropriate should such effects be observed (standard errors would likely be too small without such adjustment for site).  Among the important confounding variables not included in the analysis would indeed be exposure to violence, a factor that is often not included in similar studies, though it clearly should be if available given that depressive symptomatology is the primary independent variable here.

 In terms of additional comments, the following can be listed:

1. The data analyzed should be described as the “study sample” and not the “study population”.
2. Checking the effects of applying different cutoffs to the Edinburgh (EPDS) score is helpful from a clinical standpoint, though the intent of developing a score is to be able to identify risk that is subclinical. Hence, analyses that use the EPDS score as continuous would also be informative. Women with scores less than a cutoff are indeed not “without mental depressive symptoms”.
3. The statistical analyses did not include checks of effect modification (interaction) with depressive symptoms for salient variables on intrauterine growth.Such effects should be checked at a minimum to verify that the main effects only model is valid. Any effect modification identified would be useful in delineating the mechanism of how depressive symptoms affect intrauterine growth.
4. Very important confounders are not included in the statistical models that could alter the estimation of the effect of depressive symptoms on intrauterine growth.These would include maternal pre-pregnancy weight or BMI, as well as maternal health habits that have been shown to have associations with depressive symptoms, including maternal substance use of various kinds and the quality of prenatal care.
5. The fit of the logistic regression models with respect to calibration should include the Hosmer-Lemeshow statistic and its associated degrees of freedom and p-value. A good fitting model should have both good calibration and discrimination.
6. The discrimination abilities of the models (c statistics or area under the ROC curve) are poor and barely above the null value of 0.5.The lack of additional confounding control also likely contributed to this under-fitting. In addition, there must be some recoding of the data that somehow has resulted in c statistics less than 0.5. The authors should carefully check this. There should not be values less than 0.5. Moreover, such a coding problem has likely resulted in the stark change in the direction of the odds ratios as shown in Table 4. There should not be such a drastic change from an odds ratio of 2.18 for the EPDS cutoff of 11 that indicates higher risk of SGA to one of 0.46 for a cutoff of 0.46. This kind of error markedly reduces the confidence of the reader in the overall analysis.

I confirm that I have read this submission and believe that I have an appropriate level of expertise to confirm that it is of an acceptable scientific standard, however I have significant reservations, as outlined above.

# Response

**Published:** 2019-02-12

**Authors:** Giridhara R Babu

**Affiliations:** Public Health Foundation of India, India

**1.This paper examines the association of maternal depressive symptoms during pregnancy and small for gestation age delivery in a birth cohort in India from April 2016 to October 2017. The paper is generally well written and the tables and figures are well done and informative.**

 We sincerely thank the reviewer for the encouraging review with very constructive suggestions.

  2. **A number of points of concern, however, can be raised regarding this paper. Among these are points raised in a prior review by** **Dr.** **Desai, all of which are very pertinent. The inclusion of fetal loss deliveries would not be appropriate. If these were excluded the sample should be described as one comprised of** **livebirths** **only. Also, the inclusion of multiples would render as inappropriate analyses that assume independent observations. Not accounting for potential clustering by clinical site would additionally be inappropriate should such effects be observed (standard errors would likely be too small without such adjustment for** **site** **).**

 Thank you for the very useful comment. We have provided the responses for each point.

- Twin deliveries and stillbirths were excluded from the study analysis. We have now mentioned this in the Methods. (Page 6  and Line 10)
- Women with Multiple viable wombs are excluded from the study and analysis
- We have conducted the study in only one hospital. Therefore, there is no possibility of errors induced due to clustering.

**3** **. Among** **the important confounding variables not included in the analysis would indeed be** **exposure** **to violence, a factor that is often not included in similar studies, though it clearly should be if available given that depressive symptomatology is the primary independent variable here.**

 We understand and agree that exposure to domestic violence was not measured in our study. However, the assessment of the psychosocial environment in the pregnant women was clearly directed **at the end result of many factors **resulting in stress/depression in pregnant women such as domestic violence might have resulted in. For example, if the women is a victim of domestic violence, the questions in the questionnaire would definitely indicate that she would not have slept well or felt low or has suicidal tendencies etc. Including the assessment of domestic violence as an antecedent was not done as it would have amounted to include other sources of maternal stress/depression such as job stress, social settings, poverty etc.

**In terms of additional comments, the following can be listed:**

**4.The data analyzed should be described as the “study sample” and not the “study population”.**

 Thank you for the comment, we have made the necessary change.

**5.Checking the effects of applying different cutoffs to the Edinburgh (EPDS) score is helpful from a clinical standpoint, though the intent of developing a score is to be able to identify** **risk** **that is subclinical. Hence, analyses that use the EPDS score as continuous would also be informative. Women with scores less than a cutoff are indeed not “without mental depressive symptoms”.**

 We sincerely appreciate this comment and do agree that it is useful to examine the risk of a sub-clinical group. In this regard, we have provided a graph indicating the relation between EPDS as a continuous variable and the proportion of women delivered with SGA. ( **Supplementary File: Figure 1, Page 2**)

**6.The statistical analyses did not include checks of effect modification (interaction) with depressive symptoms for salient variables on intrauterine growth. Such effects should be checked at a minimum to verify that the main effects only model is valid. Any effect modification identified would be useful in delineating the mechanism of how depressive symptoms affect intrauterine growth.**

 We sincerely thank for this suggestion. As per the advice, we have run separate models including interaction effect. The results are provided in ( **Supplementary File: Table 1, Page 1**)

 We considered skinfold thickness as a continuous variable and excluded BMI to avoid the problem of multicollinearity.

**7.Very important confounders are not included in the statistical models that could alter the estimation of the effect of depressive symptoms on intrauterine growth. These would include maternal pre-pregnancy weight or BMI, as well as maternal health habits that have been shown to have associations with depressive symptoms, including maternal substance use of various kinds and the quality of prenatal care.**

 We have not measured the maternal pre-pregnancy weight, however, have adjusted for the maternal sum of skinfold thickness. Maternal substance use is very minimal (less than 1%) in the study sample, we have adjusted for the husband’s current tobacco and alcohol consumption.

**8.The fit of the logistic regression models with respect to calibration should include the Hosmer-Lemeshow statistic and its associated degrees of freedom and p-value. A good fitting model should have both good calibration and discrimination. The discrimination abilities of the models (c statistics or area under the ROC curve) are poor and barely above the null value of 0.5** **.The** **lack of additional confounding control also likely contributed to this under-fitting. In addition, there must be some recoding of the data that somehow has resulted in c statistics less than 0.5. The authors should carefully check this. There should not be** **values** **less than 0.5. Moreover, such a coding problem has likely resulted in the stark change in the direction of the odds ratios as shown in Table 4. There should not be such a drastic change from an odds ratio of 2.18 for the EPDS cutoff of 11 that indicates** **higher** **risk of SGA to one of 0.46 for a cutoff of 0.46. This kind of error markedly reduces the confidence of the reader in the overall analysis.**

 Thank you for pointing out this. We sincerely thank you for pointing to the error; it is very useful insight and we realized that there was a mistake in coding the variable (EPDS score cut off 11, 12, 13). We recoded the entire data set and have thoroughly checked the entire analysis after redoing it. The resulted OR changes gradually from one cut off category to other and the AUROC curves obtained from the predicted probabilities of each model are above the null value. We sincerely apologize for the mistake. Hosmer-Lemeshow test statistic indicated model is a good fit. Overall model predictability is 83.6% for EPDS cut off category 11. We tried performing discriminant analysis, but the factors found to have a significant deviation from the multivariate normal distribution.

# Peer review: Reviewer response for version 1

**DOI:** 10.21956/wellcomeopenres.15915.r33687

**Published:** 2018-09-03

**Authors:** Geetha Desai

**Affiliations:** 1 Department of Psychiatry , National Institute of Mental Health and Neuro Sciences (NIMHANS), Bengaluru, India

It is well written report. Few clarifications may be added to methods. EPDS is a self rated instrument, how was it administered to women who could not rate the tool due to illiteracy. How was the tool translated?

 Please mention that there are different cut offs that have been established for different samples (Shrestha et al. 2016 ^1^)

 In the flow chart, can you make it clear on how many had delivered when this report was written (was it 763?) or were there any exclusions due to fetal loss or twins?

 Since there is a mention of women being referred to psychiatrist if the score was more than >13 , is there a possibility that they took treatment and hence there was no link to SGA? Can you describe the public hospital, was it just one or many centers?

 Was violence assessed? As it is considered a risk factor.

 Since many of the public hospitals do not have adequate space, how was privacy ensured?

 Did any of the women have hyperemesis?

I confirm that I have read this submission and believe that I have an appropriate level of expertise to confirm that it is of an acceptable scientific standard.

## References

[1] : Reliability and validity of the Edinburgh Postnatal Depression Scale (EPDS) for detecting perinatal common mental disorders (PCMDs) among women in low-and lower-middle-income countries: a systematic review. *BMC Pregnancy Childbirth*.2016;16: 10.1186/s12884-016-0859-2 72 10.1186/s12884-016-0859-2 27044437PMC4820998

[2] : Validation of the Edinburgh Postnatal Depression Scale (EPDS) on the Thai-Myanmar border. *Trop Doct*.2017;47(4) : 10.1177/0049475517717635 339-347 10.1177/0049475517717635 28699396PMC5613805

# Response

**Published:** 2019-02-12

**Authors:** Giridhara R Babu

**Affiliations:** Public Health Foundation of India, India

1.It is a well-written report. Few clarifications may be added to methods.

 Many thanks for the encouraging review.

 2. EPDS is a self-rated instrument, how was it administered to women who could not rate the tool due to illiteracy. How was the tool translated? 

 EPDS tool was translated into the local language (Kannada) and then back-translated to English for accuracy. Through this, efforts were made to ensure a clear and conceptually accurate translation that was easily understood by the local population. The Questionnaire was then administered to the respondents by trained Research Assistants who would interview without altering the actual meaning. The response score is quantified by asking the frequency of occurrence of depressive symptoms for the number of days.

 3. Please mention that there are different cutoffs that have been established for different samples (Shrestha et al. 2016 ^1^)Thank you for this comment. We have included this in the manuscript now. (Page 5, Line 32)

 4. In the flow chart, can you make it clear on how many had delivered when this report was written (was it 763?) or were there any exclusions due to fetal loss or twins?

 Five cases were excluded as it was a twin delivery and there were four stillbirths. We have updated the flow chart.

 5. Since there is a mention of women being referred to a psychiatrist if the score was more than > 13, is there a possibility that they took treatment and hence there was no link to SGA? Can you describe the public hospital, was it just one or many centres? 

 We have referred the women with a higher score to the psychiatrist, but we have not tracked them to ascertain the treatment that they may have received. There may be a chance that they have approached a specialist and have taken treatment. Jayanagar General Hospital; a secondary level public hospital was chosen to conduct this study.

 6. Was violence assessed? As it is considered a risk factor.

 No, violence was not assessed as part of this study. We have mentioned this under the limitations now.

 7. Since many of the public hospitals do not have adequate space, how was privacy ensured?

 We thank the reviewer for this rightful concern. The research team is allotted a separate room for administering the interview and carrying out other research activities at the hospital. Thereby, efforts are consciously made to ensure that the privacy of the respondents is assured during the interviews.

 8. Did any of the women have hyperemesis?

 Seven women had hyperemesis in the study sample.
